# Supplementary material for: High-Pressure Synthesis of Cubic ZnO and Its Solid Solutions with MgO Doped with Li, Na, and K
Source: Materials (Basel). 2023 Jul 29;16(15):5341. doi: 10.3390/ma16155341 (PMC10420218; doi:10.3390/ma16155341)
Supplement: Supplementary file 1 [file materials-16-05341-s001.zip › materials-2508157-supplementary.pdf]

materials

MDPI

Supplementary materials

High-Pressure Synthesis of Cubic ZnO and Its Solid Solutions with MgO Doped with Li, Na, and K

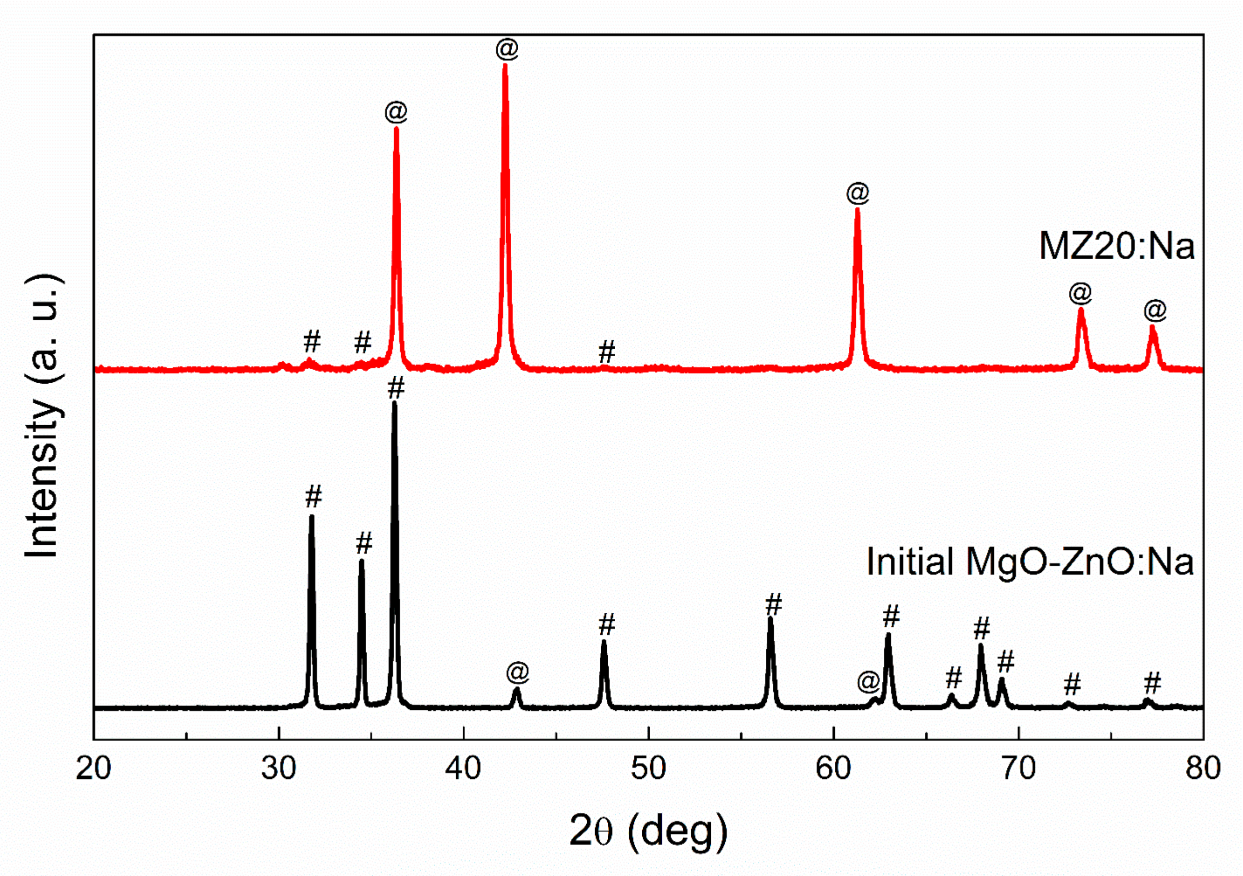

Figure S1. X-ray diffraction patterns of MZ20:Na and initial ZnO-MgO:Na mixture. (#) denotes w-ZnO phase and (@) denotes cubic pure MgO or $M_xZn_{1-x}O$:Na phase.

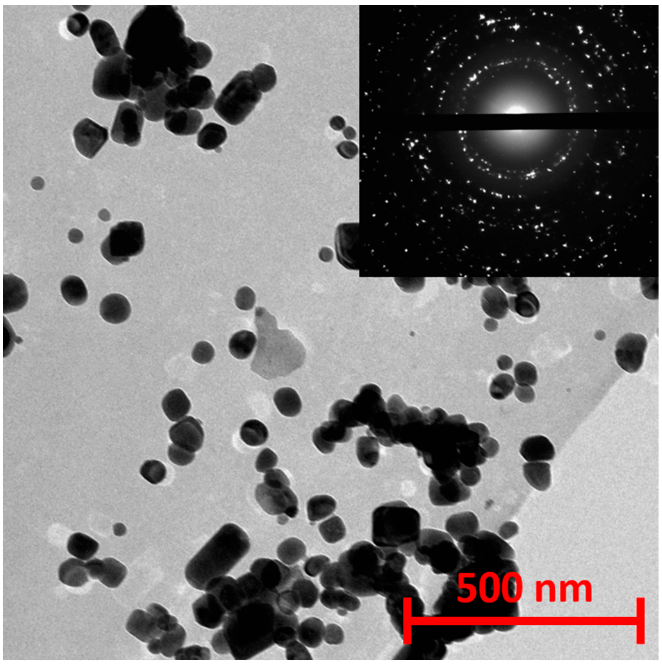

Figure S2. TEM image of w-ZnO:Li precursor with electron diffraction pattern (inset).

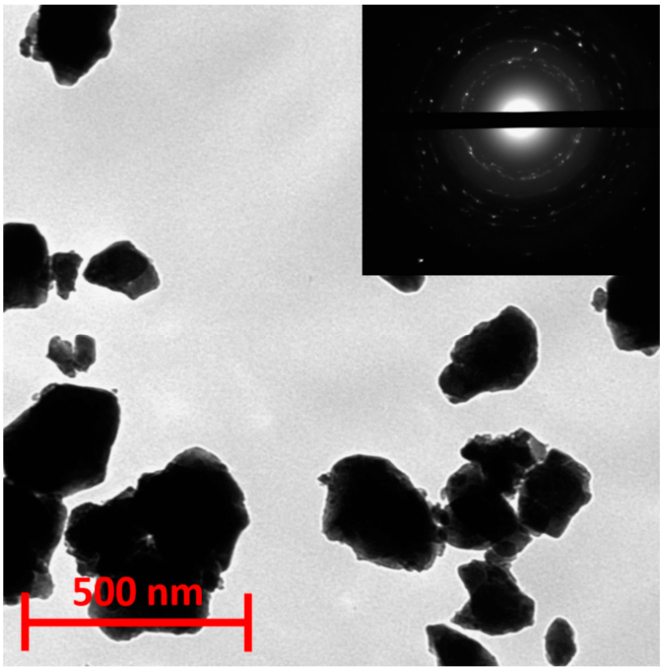

Figure S3. TEM image of rs-ZnO:Li with electron diffraction pattern (inset).

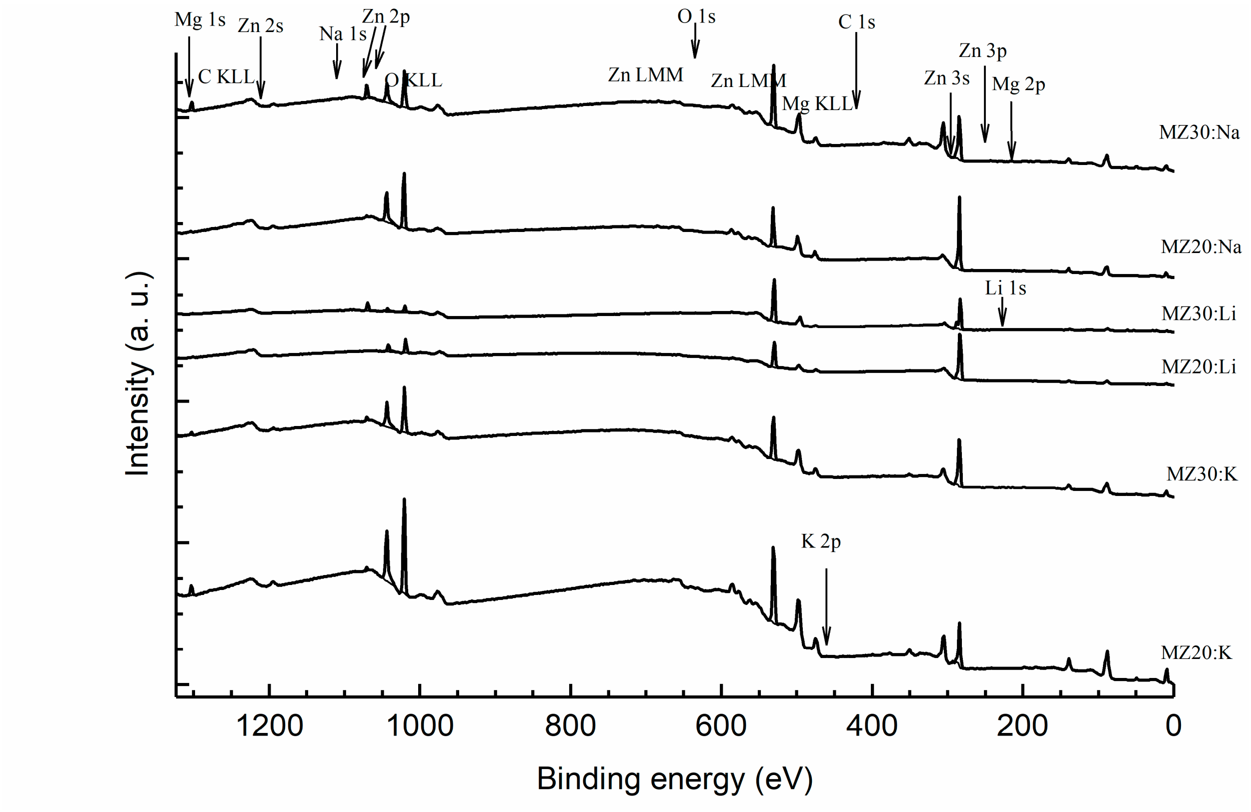

Figure S4. Overview of XPS spectra of MZ20:X and MZ30:X samples.
